# Preclinical Targeted α- and β^−^-Radionuclide Therapy in HER2-Positive Brain Metastasis Using Camelid Single-Domain Antibodies

**DOI:** 10.3390/cancers12041017

**Published:** 2020-04-21

**Authors:** Janik Puttemans, Yana Dekempeneer, Jos L. Eersels, Heleen Hanssens, Pieterjan Debie, Marleen Keyaerts, Albert D. Windhorst, Frank van der Aa, Quentin Lecocq, Karine Breckpot, Alfred Morgenstern, Frank Bruchertseifer, Tony Lahoutte, Nick Devoogdt, Matthias D’Huyvetter

**Affiliations:** 1 In vivo Cellular and Molecular Imaging laboratory, Vrije Universiteit Brussel, 1090 Brussels, Belgiumndevoogd@vub.be (N.D.); matthias.dhuyvetter@vub.be (M.D.); 2 Nuclear Medicine Department, UZ Brussel, 1090 Brussels, Belgium; 3 Amsterdam UMC (Universitair Medische Centra), Department of Radiology & Nuclear Medicine, Cancer Center Amsterdam, VU University, 1081 HV Amsterdam, The Netherlands; 4 Laboratory for Molecular and Cellular Therapy, Vrije Universiteit Brussel, 1090 Brussels, Belgium; 5 European Commission, Joint Research Centre, Department for Nuclear Safety and Security, P.O. Box 2340, 76125 Karlsruhe, Germany

**Keywords:** single-domain antibody fragment, targeted radionuclide therapy, brain metastasis, HER2

## Abstract

HER2-targeted therapies have drastically improved the outcome for breast cancer patients. However, when metastasis to the brain is involved, current strategies fail to hold up to the same promise. Camelid single-domain antibody-fragments (sdAbs) have been demonstrated to possess favorable properties for detecting and treating cancerous lesions in vivo using different radiolabeling methods. Here we evaluate the anti-HER2 sdAb 2Rs15d, coupled to diagnostic γ- and therapeutic α- and β^−^-emitting radionuclides for the detection and treatment of HER2^pos^ brain lesions in a preclinical setting. 2Rs15d was radiolabeled with ^111^In, ^225^Ac and ^131^I using DTPA- and DOTA-based bifunctional chelators and Sn-precursor of SGMIB respectively and evaluated in orthotopic tumor-bearing athymic nude mice. Therapeutic efficacy as well as systemic toxicity were determined for ^131^I- and ^225^Ac-labeled sdAbs and compared to anti-HER2 monoclonal antibody (mAb) trastuzumab in two different HER2^pos^ tumor models. Radiolabeled 2Rs15d showed high and specific tumor uptake in both HER2^pos^ SK-OV-3-Luc-IP1 and HER2^pos^ MDA-MB-231Br brain lesions, whereas radiolabeled trastuzumab was unable to accumulate in intracranial SK-OV-3-Luc-IP1 tumors. Administration of [^131^I]-2Rs15d and [^225^Ac]-2Rs15d alone and in combination with trastuzumab showed a significant increase in median survival in 2 tumor models that remained largely unresponsive to trastuzumab treatment alone. Histopathological analysis revealed no significant early toxicity. Radiolabeled sdAbs prove to be promising vehicles for molecular imaging and targeted radionuclide therapy of metastatic lesions in the brain. These data demonstrate the potential of radiolabeled sdAbs as a valuable add-on treatment option for patients with difficult-to-treat HER2^pos^ metastatic cancer.

## 1. Introduction

To this day metastasis to the brain is still a death sentence for breast cancer patients with otherwise treatable primary tumor types. Therapeutic options for secondary brain tumors are limited as traditional treatments are often ineffective within the central nervous system. As management and eradication of primary systemic tumors improve over time, patients with prolonged overall survival fall victim to disseminated cancer cells that nest within the brain. The advent of metastatic brain invasion is followed by a significant deterioration of the patient’s quality of life and reduces life expectancy to mere months after diagnosis. This makes brain metastasis an end stage in disease progression [1].

Brain metastases from systemic tumors are more prevalent than malignant primary brain tumors, and approximately 20% are caused by breast cancer [2]. In recent years there has been a steadily increasing incidence of brain metastasis, partly due to advanced imaging modalities leading to earlier detection, as well as emerging targeted treatments resulting in prolongation of survival from the primary tumor [3,4]. Unfortunately, the targeted treatments that control systemic disease are largely ineffective against brain lesions. The impermeability of the blood-brain barrier (BBB) creates a sanctuary site for micro-metastatic disease, limiting the access of large or hydrophilic therapeutics such as monoclonal antibodies (mAbs) like trastuzumab and pertuzumab in the central nervous system (CNS) [5]. Trastuzumab—a humanized mAb directed against HER2—was the first clinically-approved targeted therapeutic for the first-line treatment of HER2^pos^ breast cancer [6]. In the age before trastuzumab availability, HER2^pos^ cancers had a threefold increased probability to metastasize to the lungs, liver, and brain compared with HER2^neg^ cancers [7]. However, not all patients initially respond to trastuzumab treatment, and those that do almost universally develop resistance over time [8]. After trastuzumab or other HER2-directed treatments, the CNS becomes a highly favorable site of first recurrence for HER2-expressing cancer cells [2,9,10]. The combination of a well-treatable primary tumor type with a high potential for CNS metastasis, and therapeutics that are unable to adequately reach the CNS, is the reason for brain metastasis becoming one of the major clinical challenges. To address this need, there has been a shift in drug development towards smaller targeting compounds with better tissue penetration and higher lethality towards cancer cells [11,12]. In this regard, single-domain antibodies (sdAbs), the antigen-binding fragments of camelid heavy-chain-only antibodies, have emerged as valuable targeting vehicles for intracranial targets due to their small size (10–15 kDa), high affinity, and favorable pharmacokinetics and tissue penetration [13]. Coupled to various radionuclides, sdAbs offer fast contrast for nuclear diagnostics, and minimal accumulation in non-target organs for radionuclide therapy due to their rapid clearance from blood circulation. A phase I study using a [^68^Ga]-labeled anti-HER2 sdAb has been concluded for PET/CT assessment of HER2 expression in breast carcinoma, and a phase II trial is currently ongoing with the same tracer for characterization of HER2 expression in brain metastases of breast cancer patients [14,15]. Furthermore, the therapeutic potential of radiolabeled sdAbs has been demonstrated preclinically in various tumor types using different β^−^-particle emitting radionuclides, such as ^177^Lu and ^131^I [16,17,18]. However, β^−^-particles carry only a limited amount of ionizing energy, which they deposit over a distance of several millimeters. This low linear energy transfer (LET) makes them a less preferred choice for micrometastatic lesions, or cancer types located in highly radiosensitive tissues, such as bone marrow. Over the years, there has been a growing interest towards α−particle emitting radionuclides, due to their lethal potential for double-strand DNA damage in target cells and their short travel distance of only a few cell diameters, limiting their off-target toxicity [19]. To this end, the 2Rs15d sdAb has been evaluated in vitro and in vivo after labeling with ^225^Ac and ^211^At [20,21]. 

Here we describe the use of the anti-HER2 sdAb 2Rs15d, coupled to ^111^In or ^131^I for detection via µSPECT/CT, and coupled to ^131^I or ^225^Ac for targeted radionuclide therapy (TRNT) of HER2^pos^ brain lesions and compare its therapeutic efficacy and systemic toxicity to that of trastuzumab, a clinically-approved anti-HER2 treatment. Since 2Rs15d interacts with another HER2-domain as trastuzumab [16,22], the radiolabeled sdAb can be administered as an add-on therapy to avoid or combat the onset of trastuzumab-resistance or the formation of micrometastatic lesions in immunoprivileged sites, such as the brain. The integration of molecular imaging in case of the theranostic radionuclide ^131^I was included to predict therapeutic efficacy during treatment.

## 2. Results

### 2.1. Different HER2^pos^ Cell Lines React Differently In Vitro to Treatment with Trastuzumab

**Figure 1:**
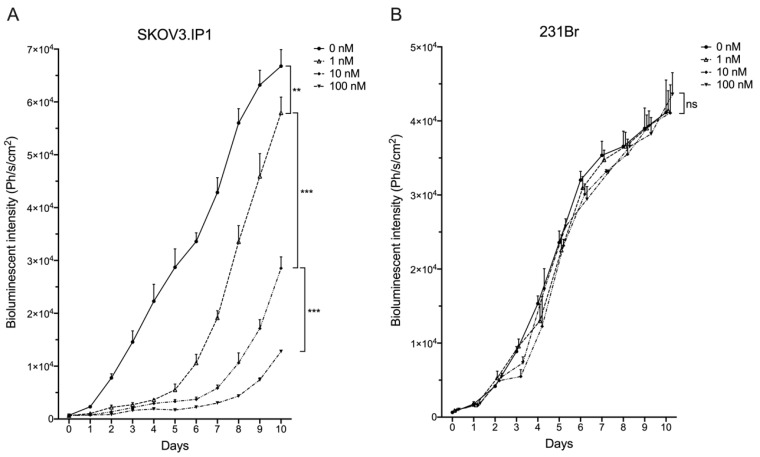
In vitro trastuzumab-mediated growth inhibition of (**A**) SKOV3.IP1 cells, naturally expressing high HER2 levels and (**B**) 231Br cells, stably transfected to express high levels of HER2. Cells were cultured with different trastuzumab concentrations from day 0–5, followed by trastuzumab-free medium at day 6–10. Concentration-dependent growth inhibition can be observed for SKOV3.IP1 cells during trastuzumab treatment. 231Br cell growth was not hampered, regardless of trastuzumab concentration. (ns: not significant, ** *p* < 0.01, *** *p* < 0.005).

The HER2^pos^ and firefly luciferase (Fluc)^pos^ human ovarian cancer cell line SK-OV-3-Luc-IP1 (further referred to as SKOV3.IP1) and HER2^pos^/Fluc^pos^ human breast carcinoma cell line MDA-MB-231Br (further referred to as 231Br) were exposed in vitro to different concentrations of trastuzumab. Cell growth was correlated with bioluminescence after addition of D-luciferin. Five days after being challenged with trastuzumab-enriched medium, cells were allowed to grow in Trastuzumab-free growth medium. SKOV3.IP1 cells showed a concentration-dependent decrease in bioluminescent signal upon trastuzumab incubation (Figure 1A). Contrarily, 231Br cells were not growth inhibited by trastuzumab, regardless of concentration (Figure 1B). So, SKOV3.IP1 cells represent a trastuzumab-sensitive model whereas 231Br cell are trastuzumab-resistant.

### 2.2. Intracranially Inoculated HER2^pos^ Cells Show Aggressive Exponential Growth In Vivo

**Figure 2:**
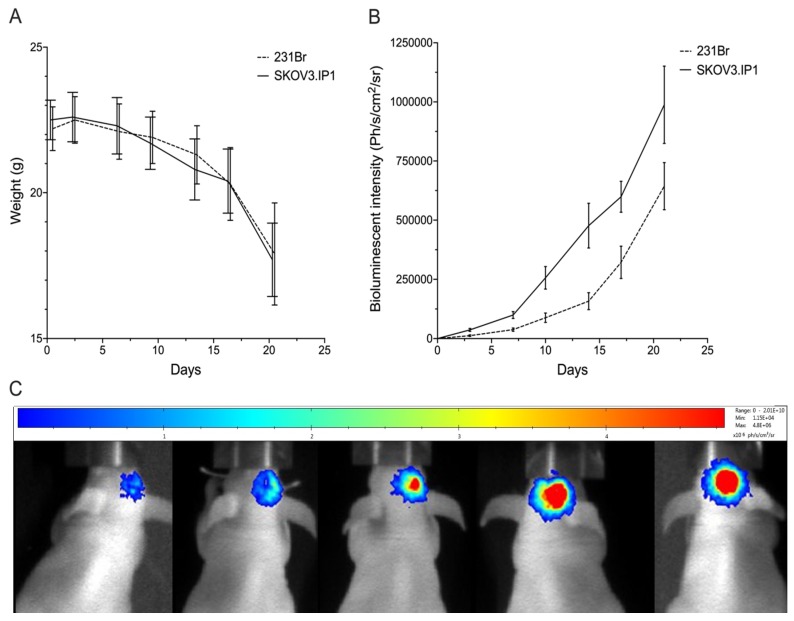
(**A**) Follow-up of SKOV3.IP1 an 231Br tumor growth in function of time using body weight measurements, and (**B**) quantitative (photons/s/cm^2^/steradian) and (**C**) visual in vivo bioluminescence imaging at day 3, 7, 10, 14, 17 and 21 post-inoculation (images shown of SKOV3.IP1 tumors and are representative for both tumor models). Values are presented as mean ± SD.

Athymic nude mice were inoculated intracranially with SKOV3.IP1 or 231Br cells. Weight loss was minimal during the first 17 days after inoculation, before onset of significant weight loss occurred around day 21 (Figure 2A). Tumor growth was monitored using in vivo BLI after intraperitoneal injection of D-luciferin (Figure 2B,C). Both SKOV3.IP1 and 231Br orthotopic tumors show exponential growth characteristics. 

### 2.3. SdAbs Can Be Labeled Efficiently with Different Radionuclides

SdAbs and Trastuzumab were conjugated with *p*-SCN-Bn-CHX-A″-DTPA for ^111^In-labeling and conjugated with *p*-SCN-Bn-DOTA for ^225^Ac-labeling. After ^111^In-radiolabeling, iTLC revealed radiochemical purities of 91.3 ± 2.1% and 99.1 ± 0.4%, before and after SEC purification, respectively. 

24 h after ^225^Ac-radiolabeling, iTLC revealed radiochemical purities of 86.8 ± 2.1% and 98.1 ± 0.6%, before and after SEC purification, respectively. Radiochemical purity of [^131^I]-2Rs15d after purification was 98.1 ± 0.9% as determined via iTLC. 

### 2.4. ^111^In-Labeled Anti-HER2 sdAb Shows Favorable In Vivo Biodistribution Compared to Trastuzumab

**Figure 3:**
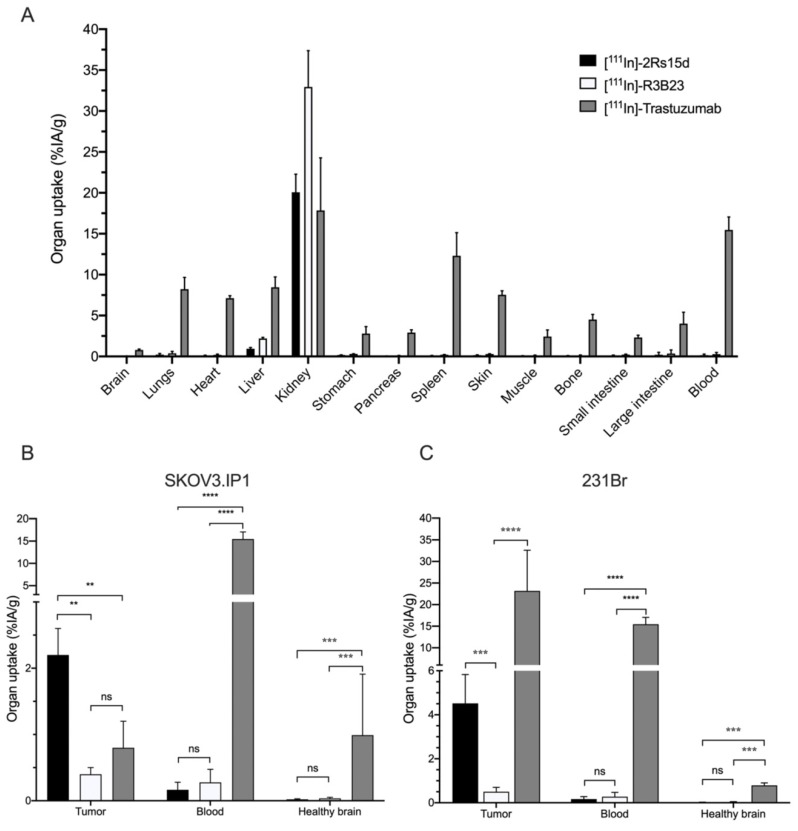
Ex vivo biodistribution of radiolabeled anti-HER2 tracers in brain tumor-bearing mice. (**A**) Ex vivo biodistribution analyses of [^111^In]-labeled compounds in HER2^pos^ tumor-bearing mice, at 1 h p.i. (sdAbs) and 3 d p.i. (mAb) (*n* = 4). For sdAbs only kidneys show elevated uptake, whereas trastuzumab has increased tracer uptake in all highly vascularized organs. Detail of tracer uptake in tumor, brain and blood shows a significantly higher tumor uptake for [^111^In]-2Rs15d in (**B**) SKOV3.IP1 and (**C**) 231Br brain tumors compared to the non-targeting [^111^In]-R3B23. Increased tumor-to-brain ratio of [^111^In]-trastuzumab was only observed in 231Br tumors (**C**). (ns: not significant, ** *p* < 0.01, *** *p* < 0.005, **** *p* < 0.001).

**Figure 4:**
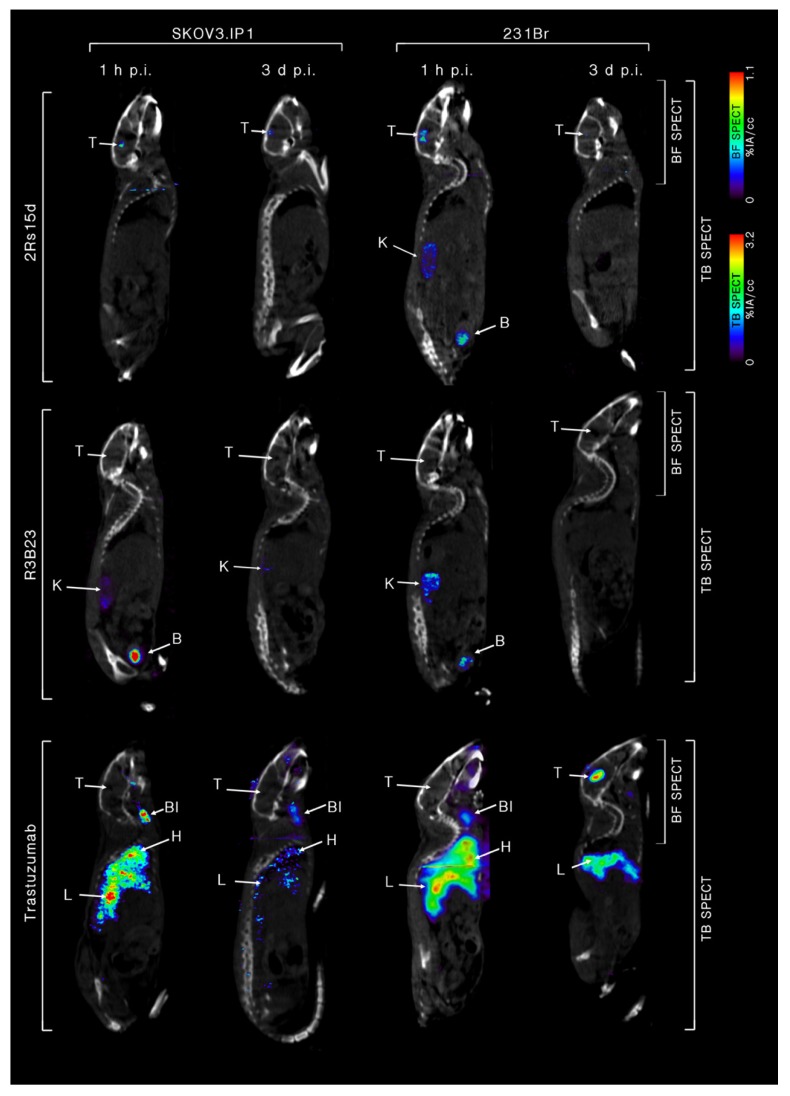
Sagittal view of fused whole-body and brain-focused µSPECT/CT scan images of anti-HER2 [^111^In]-2Rs15d, non-targeting [^111^In]-R3B23 and anti-HER2 [^111^In]-trastuzumab 1 h and 3 days p.i. in SKOV3.IP1 (**Left**) and 231Br (**Right**) tumor models. One representative image of each group (*n* = 4) is shown. Tumor-targeting is visible for [^111^In]-2Rs15d in both tumor models, whereas [^111^In]-trastuzumab was only able to accumulate in 231Br tumors. High uptake was also observed in highly vascularized organs up to 3 days after injection. There was no aspecific leakage of non-targeting [^111^In]-R3B23 into the tumor site. T: tumor, Bl: blood, K: kidney, H: heart, L: liver, B: bladder, TB: total body, BF: brain focus.

Twenty one days after intracranial inoculation, SKOV3.IP1 and 231Br tumor-bearing mice were intravenously (i.v.) injected with either ^111^In-labeled 2Rs15d, R3B23 or trastuzumab (*n* = 4). 1 h and 3 days post-injection (p.i.) mice were imaged using whole-body and brain-focused µSPECT/CT. [^111^In]-2Rs15d shows extremely low uptake in non-target organs apart from kidneys and bladder (Figure 3A) with high specific uptake in both HER2^pos^ brain tumor types as early as 1 h p.i. (Figure 3B,C). This specific accumulation is detectable within the lesions up to 3 days p.i. of the radiotracer. Non-targeting [^111^In]-R3B23 sdAb showed no aspecific accumulation within brain lesions or other organs, apart from kidneys and bladder. [^111^In]-trastuzumab shows high, slow-clearing blood pool activity 1 h and 3 days p.i., resulting in high aspecific accumulation in highly vascularized organs, such as heart, liver and spleen (Figure 3A). Importantly, high specific uptake is observed in 231Br tumors 3 days p.i. (Figure 3B), whereas there is no visible accumulation in SKOV3.IP1 tumors (Figure 3C), so the in vitro trastuzumab-sensitive SKOV3.IP1 brain metastasis model is permissive for small sdAb but not for large trastuzumab accumulation, while the in vitro trastuzumab-resistant 231Br brain metastasis model allows uptake of both sdAb and trastuzumab. These notions are confirmed by ex vivo radioactive quantification data of dissected organs: [^111^In]-2Rs15d (Figure 3B,C) showed uptake values of 2.2 ± 0.4 %IA/g and 4.52 ± 1.31 %IA/g in SKOV3.IP1 and 231Br tumors, respectively. The uptake values in other organs and tissues were below 1 %IA/g, except for kidneys. Non-targeting [^111^In]-R3B23 sdAb showed significantly lower tumor-uptake (0.4 ± 0.1 %IA/g and 0.5 ± 0.2 %IA/g for SKOV3.IP1 and 231Br tumors, respectively) (Figure 3B,C), confirming no non-specific leakage of radiotracer within brain lesions caused by disruption in the BBB. Tumor uptake of [^111^In]-trastuzumab was significantly different (*p* < 0.001) for SKOV3.IP1 and 231Br tumors (0.8 ± 0.4 %IA/g vs. 23.2 ± 9.4 %IA/g, respectively). Representative fused whole-body and brain-focused µSPECT/CT images are shown in Figure 4.

### 2.5. Dosimetry Calculations of A Single Dose [^131^I]-2Rs15d and [^225^Ac]-2Rs15d

231Br tumor-bearing mice (*n* = 3) were injected with 3.67 MBq [^131^I]-2Rs15d or 65 kBq [^225^Ac]-2Rs15d after which mice were killed 1, 4, 12, 24, 48 and 72 h post-injection. The biodistribution data of all major organs were time-integrated to calculate the residence time of [^131^I]-2Rs15d per organ. Organ-absorbed doses from therapeutic-equivalent activities of 37 MBq for [^131^I]-2Rs15d and 85 kBq for [^225^Ac]-2Rs15d are summarized in Table 1 and Table 2, respectively. Kidneys received the highest absorbed dose of 12,50 Gy and 3,49 Gy, while tumors received 4,90 Gy and 1,47 Gy for ^131^I-2Rs15d and ^225^Ac-2Rs15d respectively. Doses delivered to other healthy organs and tissues were very low, except for ^131^I-2Rs15d in the thyroid, which received about 4 Gy. 

### 2.6. [^131^I]-2Rs15d Shows Theranostic Potential for HER2^pos^ Brain Lesions

**Figure 5:**
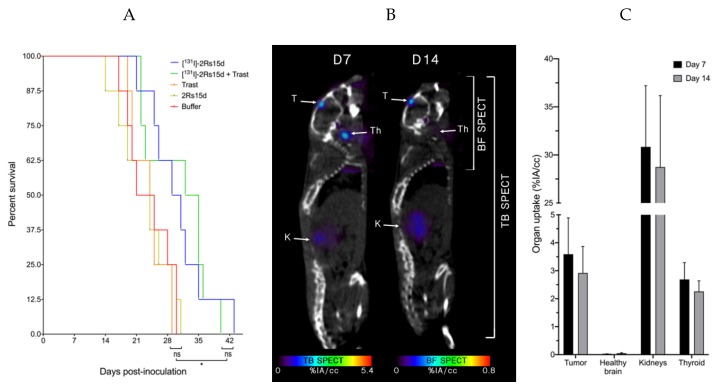
Event-free survival during TRNT. Events were defined as (i) mortality, (ii) weight loss > 20%, (iii) immobility, (iv) unresponsiveness to external stimuli. 231Br tumor-bearing mice (*n* = 8 per group, tumor inoculation on Day 0) were treated with intravenous injections of [^131^I]-2Rs15d (Day 7, 14), combination treatment of [^131^I]-2Rs15d (Day 7, 14) and trastuzumab (Loading dose; day 7-maintenance dose; day 10, 14, 17, 21, 24, 28) or trastuzumab as single agent (Loading dose; day 7-maintenance dose; day 10, 14, 17, 21, 24, 28). Control groups received either vehicle buffer or unlabeled 2Rs15d in equimolar quantities as treated groups at identical timepoints (**A**). 231Br tumor-bearing mice (*n* = 3, tumor inoculation on Day 0) received [^131^I]-2Rs15d (Day 7, 14), followed by µSPECT/CT imaging 2 h p.i. Fused µSPECT/CT images (**B**) and image quantification (**C**) showed accumulation of activity in brain tumor, thyroid and kidneys. Trast: Trastuzumab, T: tumor, K: kidney, Th: thyroid, TB: total body, BF: brain focus (ns: not significant, * *p* < 0.05).

We next investigated whether ^131^I-labeled anti-HER2 sdAb has therapeutic efficacy in the trastuzumab-resistant model. A detailed schematic timeline of the treatment regime can be found in Supplementary Figure S1. Mice bearing small 231Br intracranial tumors that received [^131^I]-2Rs15d or a combination treatment of [^131^I]-2Rs15d and trastuzumab had a significantly longer (*p* < 0.05) median survival of 30 and 33.5 days respectively versus 23 and 24.5 days for mice receiving vehicle buffer or trastuzumab, respectively. No statistically significant difference in median survival was observed between groups receiving [^131^I]-2Rs15d and the combination treatment of [^131^I]-2Rs15d and trastuzumab (*p* = 0.84). No significant difference in survival was observed between the groups receiving either vehicle buffer, unlabeled sdAb or trastuzumab as single agent (*p* > 0.62) (Figure 5A).

Subsequent imaging of mice (*n* = 3) 2 h after treatment with [^131^I]-2Rs15d confirmed uptake in the tumor lesions (3.59 ± 1.30 %IA/cc), as well as thyroid (2.69 ± 0.6 %IA/cc) and kidneys (30.84 ± 6.36 %IA/cc). Uptake in other organs was below 1 %IA/cc (Figure 5B,C).

### 2.7. SdAb-Mediated Targeted Alpha Therapy Inhibits Cell Growth in Trastuzumab-Responsive and -Resistant Tumor Models

**Figure 6:**
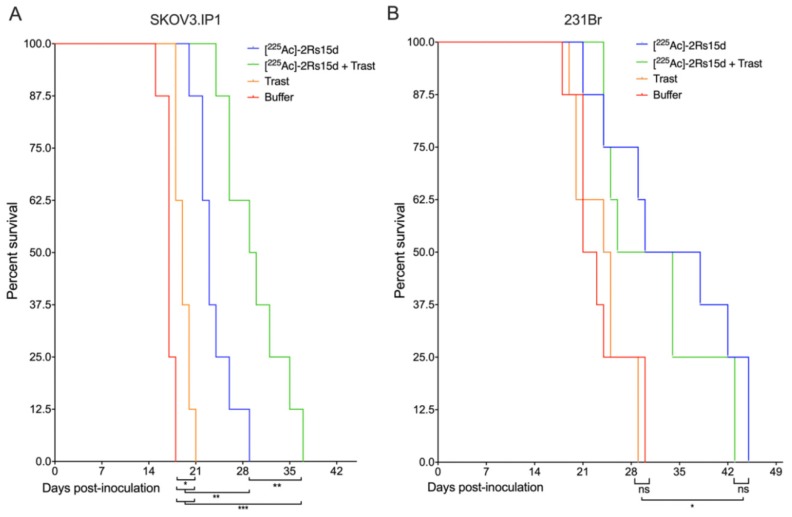
Event-free survival during targeted alpha therapy. Events were defined as (i) mortality, (ii) weight loss > 20%, (iii) immobility, (iv) unresponsiveness to external stimuli. Trastuzumab-sensitive SKOV3.IP1 (**A**) and trastuzumab-resistant 231Br (**B**) tumor-bearing mice (*n* = 8 per group, tumor inoculation on Day 0) were treated with intravenous injections of either [^225^Ac]-2Rs15d (Day 7, 10, 14), combination treatment of [^225^Ac]-2Rs15d (Day 7, 14, 21) and trastuzumab (Loading dose; day 7-maintenance dose; day 10, 14, 17, 21, 24, 28) or trastuzumab as single agent (Loading dose; day 7-maintenance dose; day 10, 14, 17, 21, 24, 28). The control group received vehicle buffer at identical timepoints as treated groups. 150 mg/kg gelofusin was co-administered with [^225^Ac]-2Rs15d in order to reduce kidney retention. (ns: not significant, * *p* < 0.05, ** *p* < 0.01, *** *p* < 0.005).

We next investigated whether besides β^−^-particle emitting sdAbs, sdAbs that are labeled with α-particle emitters can also show therapeutic efficacy for brain-metastases. A detailed schematic timeline of the treatment regime can be found in Figure S2. Mice bearing small trastuzumab-sensitive SKOV3.IP1 intracranial tumors that received [^225^Ac]-2Rs15d or a combination treatment of [^225^Ac]-2Rs15d and trastuzumab had a significantly longer (*p* < 0.001) median survival of 23 and 29.5 days respectively versus 17 days for mice receiving vehicle buffer. Animals treated with trastuzumab alone lived on average 2 days longer than the control group (*p* < 0.05) and trastuzumab in combination with [^225^Ac]-2Rs15d prolonged median survival with 6.5 days compared to those treated with [^225^Ac]-2Rs15d alone (*p* < 0.01) (Figure 6A). 

Mice bearing small trastuzumab-resistant 231Br intracranial tumors that received [^225^Ac]-2Rs15d or a combination treatment of [^225^Ac]-2Rs15d and trastuzumab had a significantly longer (*p* < 0.05) median survival of 34 and 30 days respectively versus 22 and 24.5 days for mice receiving vehicle buffer or trastuzumab, respectively. No statistically significant difference in median survival was observed between groups receiving [^225^Ac]-2Rs15d and the combination treatment of [^225^Ac]-2Rs15d and trastuzumab (*p* = 0.437). No significant difference in survival was observed between the groups receiving either vehicle buffer or trastuzumab as single agent (*p* = 0.734) (Figure 6B). 

### 2.8. [^225^Ac]- and [^131^I]-sdAb TRNT Shows No Obvious In Vivo Toxicity

Brain cross sections of tumor-bearing mice showed focal to multifocal lesions, mainly within the leptomeninges or ventricular system. Tumors presented multiple areas of necrosis, vascular invasion, and very infiltrative neoplastic growth. Two doses of 32.37 ± 1.83 MBq [^131^I]-2Rs15d or three doses of 81.67 ± 28.87 kBq [^225^Ac]-2Rs15d showed no significant signs of toxicity or mortality compared to control-treated animals during the treatment follow-up. No significant treatment-related changes in organ weight/ratios or gross pathology were observed. Only minimal multifocal, tubular dilation was observed within kidneys of [^131^I]-2Rs15d-treated mice and mild diffuse tubular dilation for [^225^Ac]-2Rs15d-treated mice. A score table summarizing most relevant toxicity severity per organ for each treatment group can be found in Table S1.

## 3. Discussion

HER2 is to this date one of the most exploited cancer markers for targeted therapies, mainly in the context of breast and gastric cancer [23]. However, determination of a patient’s HER2 status is routinely performed based on analysis of invasive biopsies. Since a single biopsy does not reflect expression heterogeneity within a single tumor, expression in metastatic lesions, or up- and downregulation of cancer markers during treatment, these techniques often fail at representing the overall status of HER2 expression [2,24]. This is especially crucial when primary tumors metastasize to the brain, where biopsies are challenging at best to perform. Therefore, non-invasive imaging modalities using diagnostic tracers targeting HER2 could prove to be essential in patient selection and monitoring of disease progression, considering 25% of patients with HER2^pos^ breast cancer will eventually develop brain metastases [25]. To this end, several anti-HER2 antibodies, antibody fragments and small molecules have been labeled with various diagnostic radionuclides for nuclear imaging [14,26,27,28,29]. A major advantage of this approach is that diagnostic radionuclides can be interchangeable with therapeutic β^−^ or α-emitting radionuclides for treatment, or ideally radionuclides that emit both β^−^ or α- as well as γ or β^+^ radiation [30,31,32,33,34]. This theranostic approach of combining the same vehicle for diagnostic imaging and TRNT can predict which patients will respond to therapy and predict therapeutic outcome during treatment. 

We recently reported the first-in-human application of ^68^Ga-radiolabeled 2Rs15d sdAb for the assessment of HER2 expression in breast carcinoma and the same compound is now under investigation for the detection of HER2^pos^ brain metastatic lesions [14,15]. A phase I study was recently concluded to evaluate safety, biodistribution, radiation dosimetry and tumor imaging potential of [^131^I]-2Rs15d in healthy volunteers and breast cancer patients. [^131^I]-2Rs15d proved to accumulate in HER2^pos^ cancer lesions, while clearing fast from non-target tissues via the kidneys. [^131^I]-2Rs15d’s favorable dosimetry would allow administration of therapeutic radioactive doses with a minimum risk of toxicity [35]. In addition, [^177^Lu]-2Rs15d has been applied preclinically for TRNT in mice with HER2^pos^ SKOV3 subcutaneous xenografts, leading to almost complete tumor regression and significantly improved median survival [17]. In a peritoneal SKOV3.IP1 metastatic model, treatment with [^131^I]-2Rs15d prolonged median survival with 20 days compared to untreated animals [16]. Ex vivo biodistribution of [^225^Ac]-2Rs15d in SKOV3 tumor-bearing mice showed high tumor uptake and exceptionally low kidney retention when coinjected with the plasma expander Gelofusin [20].

In this study we report the first theranostic application of radiolabeled sdAbs with regards to brain tumors and metastasis. 2Rs15d makes an excellent candidate due to its favorable pharmacokinetics and high affinity. Importantly, it does not compete with trastuzumab or pertuzumab, allowing for coadministration of both therapeutics, or provide an alternative treatment for patients who progress on trastuzumab or trastuzumab-DM1, which is often the case when brain metastasis occurs. Therapeutics that are effective at controlling systemic disease often fail at reaching the same results within the central nervous system, either through acquired resistance mechanisms or inaccessibility to large biomolecules [36,37,38,39]. Whether sdAbs can trespass the intact BBB remains a topic of debate. Thus far, most sdAbs with regard to cerebral applications that do not rely on BBB disruption have been in the context of neurodegenerative disease, such as Alzheimer’s disease [40,41,42] and Parkinson’s disease [43,44].

To evaluate 2Rs15d’s added value as a theranostic radiopharmaceutical and potential advantage over trastuzumab for difficult-to-treat brain tumors, two HER2-overexpressing cell lines were used, one sensitive and one resistant to trastuzumab as demonstrated in vitro. In vivo biodistribution of both ^177^Lu-labeled 2Rs15d and trastuzumab were performed previously in subcutaneous xenografted mice at different timepoints [17]. The 1 h p.i. timepoint shows the most optimal biodistribution profile regarding tumor accumulation and renal clearance for small sdAbs, whereas trastuzumab requires much longer to reach its highest uptake in target tissues and be cleared from the blood. Biodistribution of [^111^In]-2Rs15d in mice bearing either intracranial SKOV3.IP1 or 231Br tumors 21 days after inoculation showed high tumor uptake in both models 1 h p.i. (2.2 ± 0.4 %IA/g and 4.52 ± 1.31 %IA/g respectively), with very low accumulation in healthy tissue and fast clearance via the kidneys. This corresponds well with previously obtained results regarding [^131^I]-2Rs15d uptake in subcutaneous SKOV3 and BT474/M1 xenografts (2.31 ± 0.22 %IA/cc and 6.48 ± 2.58 %IA/cc respectively) [16]. Zhou et al. describes the use of ^18^F-labeled anti-HER2 sdAbs 2Rs15d and 5F7 for PET imaging of HER2^pos^ intracranial BT474/M1Br-Fluc tumors, reaching 6.4 %IA/g 1 h p.i. (*n* = 1) [45]. The increased uptake in BT474/M1 tumors can be explained by their remarkably high HER2-expression profile. The absence of accumulation of non-targeting sdAb [^111^In]-R3B23 in both models demonstrates the specificity of the anti-HER2 sdAb. [^111^In]-trastuzumab was only able to accumulate in 231Br tumors, with the highest uptake 3 days post-injection. Since the 231Br cell line is optimized for metastasis to the brain, it exhibits a strong invasive behavior which potentially leads to a more disrupted BBB, making lesions more accessible to large biomolecules [46]. SKOV3.IP1 was optimized for intraperitoneal growth via in vivo maturation, and lesions showed better demarcated regions in the brain, with a low degree of vascularization. It is noteworthy that the uptake of trastuzumab (23.2 ± 9.4 %IA/g) was significantly higher in 231Br tumors than was the case for 2Rs15d (4.52 ± 1.31 %IA/g). This correlates well with tumor uptake of [^177^Lu]-trastuzumab in HER2^pos^ SKOV3 subcutaneous xenografted mice (22.85 ± 4.24 %IA/g) [17]. In case of substantial BBB disruption, the mAb benefits from a longer circulation time and higher avidity compared to the sdAb, leading to a higher retention within the target lesion. Terrell-Hal and colleagues also demonstrated [^125^I]-Trastuzumab’s ability to cross the disrupted BBB with ~5% of injected dose reaching HER2^pos^ MDA-MB 231Br brain tumors [47]. Longer circulation in the blood also implies that non-target organs are exposed to radiation for a longer time, resulting in slower high-contrast imaging and a higher off-target toxicity profile. PET-imaging of HER2^pos^ metastatic breast cancer patients using [^89^Zr]-trastuzumab 4-5 days p.i. showed relative uptake values (RUVs) (mean ± SEM) of 3.5 ± 4.2 in brain lesions, compared to 5.9 ± 2.4, 2.8 ± 0.7, 4.0 ± 0.7, and 0.20 ± 0.1 in liver, spleen, kidneys, and brain tissue, respectively [28]. 

Next, sdAb-based targeted radionuclide therapy was performed in HER2^pos^ 231Br tumor bearing mice 7 days after inoculation as a preliminary model to mimic minimal residual or micrometastatic disease. The therapeutic efficacy of [^131^I]-2Rs15d was compared to trastuzumab as single agent, as well as the combination treatment of [^131^I]-2Rs15d and trastuzumab. Control groups included mice treated with either the vehicle buffer or unlabeled 2Rs15d sdAb. Mice treated with [^131^I]-2Rs15d showed a significant increase in median survival of 30 and 33.5 days when administered as single agent or combination treatment respectively. The addition of trastuzumab had no effect on survival, which confirms the data obtained from the in vitro inhibition assay. Subsequent µSPECT/CT imaging of mice treated with [^131^I]-2Rs15d confirms specific uptake within the tumor lesion, as well as kidneys and thyroid. Importantly, since 2Rs15d does not compete with trastuzumab for epitope binding, this theranostic application is still valuable when co-administrating 2Rs15d and trastuzumab. Dosimetry based on ex vivo biodistribution data confirmed most activity is retained within the kidneys until 48 h after administration, however after extrapolation to therapeutic activities the cumulative absorbed dose (±25 Gy) remains close to the considered toxicity threshold of 23 Gy to kidneys. However, this threshold was determined using fractionated external beam radiotherapy, and does not fully represent a systemic targeted treatment to predict the risk of organ failure [48]. No short-term kidney toxicity was observed after administering ±65 MBq of [^131^I]-2Rs15d. Accumulation of free ^131^I in the thyroid could be circumvented by administering cold iodine before administering radioiodinated tracers. Indeed, in the phase I trial by Keyaerts et al. there was no accumulation of activity detected after [^131^I]-2Rs15d administration in patients that received potassium iodide daily for several consecutive days starting 24 h before the administration of [^131^I]-2Rs15d [35]. 

Recent clinical trials exploring targeted treatments based on α-emitting isotopes show the improved efficacy and reduced toxicity of these radionuclides [49,50,51,52]. Of potential therapeutic α-emitters, ^225^Ac offers a good balance between ease-of-labeling, practical half-life of 10 days, four α-particles per decay and isotope availability. It has been shown recently that 2Rs15d can be labeled with ^225^Ac via DOTA chelation and maintains its affinity and pharmacokinetic profile. [^225^Ac]-2Rs15d showed a fast uptake in SKOV3 tumors compared to HER2^neg^ MDA-MB-231 (4.01 ± 1.58 %IA/g vs. 0.49 ± 0.20 %IA/g 2 h p.i.), with high tumor-to-tissue ratios. In addition, coadministration of Gelofusin with [^225^Ac]-2Rs15d reduced kidney retention 3-fold compared to [^225^Ac]-2Rs15d alone [20]. In this study, we implemented the use of [^225^Ac]-2Rs15d for targeted α-therapy in SKOV3.IP1 and 231Br brain tumor-bearing mice. Dosimetry calculations based on ex vivo biodistribution confirmed accumulation of [^225^Ac]-2Rs15d mainly in tumor and kidneys, however the absorbed dose is considerably lower than for the ^131^I-labeled sdAb, allowing for an additional administration of [^225^Ac]-2Rs15d. Mice were treated with either [^225^Ac]-2Rs15d, trastuzumab, a combination of [^225^Ac]-2Rs15d and trastuzumab, or vehicle buffer. Reduced kidney retention was partially achieved by coadministrating 150 mg/kg gelofusin. For 231Br tumor-bearing mice, results were comparable with [^131^I]-2Rs15d-treated mice. [^225^Ac]- 2Rs15d led to an increase in median survival of 12 days compared to the control group, whereas [^131^I]-2Rs15d extended median survival by 10.5 days. The comparable result in therapeutic efficacy despite the lower absorbed dose is most likely due to the higher relative biological effectiveness of α-particles compared to β^−^-particles [53]. Trastuzumab had no effect on therapeutic outcome as single agent or add-on treatment. It is important to note that in immune-deficient mice, trastuzumab does not fully benefit from the host’s immune response which plays a key role in the anti-tumor activity on top of inhibiting downstream signaling by preventing receptor dimerization [54]. 

For SKOV3.IP1 tumor-bearing mice, trastuzumab prolonged survival significantly compared to controls (19 vs. 17 days, *p* < 0.05). [^225^Ac]-2Rs15d prolonged median survival by 6 days (23 vs. 17 days, *p* < 0.01), whereas the combination with trastuzumab led to an increase of 12.5 days (29.5 vs. 17, *p* < 0.001). Interestingly, despite the low uptake of [^111^In]-trastuzumab in SKOV3.IP1 brain tumors, there is still a therapeutic effect noticeable, in particular in combination with [^225^Ac]-2Rs15d. This could imply that either trastuzumab is still very cytotoxic at extremely low doses or coupling to DTPA decreased its ability to permeate through SKOV3.IP1 brain lesions. The success of the combination treatment in case of SKOV3.IP1 tumors could be the result of α-particle-enhanced BBB permeabilization of [^225^Ac]-2Rs15d, causing a leaky blood-brain- or blood-tumor-barrier, allowing access to larger molecules such as mAbs [55,56]. This synergistic effect was previously observed in mice bearing intraperitoneal SKOV3.IP1 tumors. Animals receiving trastuzumab in combination with [^131^I]-2Rs15d lived on average 50 days longer than the controls, and on average 30 days longer than those treated with [^131^I]-2Rs15d alone [16]. These results are in line with the improved prognosis of HER2^pos^ cancer patients, where the advent trastuzumab has drastically enhanced the outcome in case of primary breast cancer with or without systemic metastases. However, when metastasis to the brain is involved, the antibody fails to hold up to the same promise. The limited therapeutic success of trastuzumab in brain tumors compared to systemic tumors emphasizes the need for alternative treatments.

In total, these results demonstrate that sdAbs can be valuable vehicles for a theranostic approach to detect and combat brain lesions, as single agent or as an add-on therapy for treatment-resistant cancers. It is important to note that these tumor models are very aggressive and are more likely to be fatal in the early stages of disease progression compared to subcutaneous or intraperitoneal tumors. Yet, we described significant survival benefits when using the anti-HER2 sdAb 2Rs15d, either coupled to ^225^Ac or ^131^I in 2 models where trastuzumab, the clinical standard for HER2-targeted treatment, shows to be largely ineffective.

## 4. Materials and Methods

### 4.1. General

All reagents used in cell culture experiments were purchased from Gibco BRL (Grand Island, NY, USA) except when noted. All other reagents were purchased from Sigma-Aldrich (Darmstadt, Germany) except where otherwise noted. Anti-HER2 sdAb 2Rs15d and non-targeting sdAb R3B23 were generated as described previously [22,57]. Trastuzumab (Herceptin^®^, Genentech, San Francisco, CA, USA) was used as stated in the experiments.

### 4.2. Cell Culture Conditions

The HER2^pos^ and firefly luciferase (Fluc)^pos^ human ovarian cancer cell line SK-OV-3-Luc-IP1 (SKOV3.IP1) was a kind gift of prof. Marc Bracke (Ghent University, Ghent, Belgium) [58]. SKOV3.IP1 cells were cultured in supplemented DMEM. The HER2-GFP-transfected human breast carcinoma MDA-MB-231Br—a modified subclone of naturally HER2^neg^ MDA-MB-231 optimized for metastasis to the brain—was a kind gift from Patricia Steeg (Center for Cancer Research, National Cancer Institute, Bethesda, MD, USA) [46]. HER2^pos^/GFP^pos^ MDA-MB-231Br were transduced in-house using lentiviral vectors produced with the lentiviral transfer plasmid pSINT-CMV-FLUC, as described previously [59,60]. HER2^pos^, GFP^pos^ and Fluc^pos^ MDA-MB-231Br (231Br) cells were cultured in supplemented DMEM containing 200 µg/mL Zeocin (Invitrogen, Paisley, UK).

All media were enriched with 10% fetal bovine serum, and a mixture of 100 U/mL penicillin and 0.1 mg/mL streptomycin (Invitrogen). Cells were grown in a humidified atmosphere with 5% CO_2_ at 37 °C. Prior to use for in vitro and in vivo purposes, cells were detached using TrypLE Express. 

### 4.3. In Vitro Trastuzumab-Induced Growth Inhibition of HER2-Expressing Cells

5 × 10^3^ SKOV3.IP1and 231Br cells per well were adhered overnight in a 6-well plate and washed 2 times with PBS prior to addition of 0, 1, 10, 50 and 100 nmol/L trastuzumab. Bioluminescence was measured daily after addition of 1 µg D-luciferin (Promega, Leiden, The Netherlands) using a Photon Imager device (Biospace Lab, Nesles la Vallée, France). After five days, cells were washed twice with PBS and replenished with trastuzumab-free growth medium. Bioluminescence was measured daily as described before until 95–100% confluency was reached.

### 4.4. Preparation of Radiolabeled Compounds

Anti-HER2 sdAb 2Rs15d, anti-HER2 mAb trastuzumab, and non-targeting sdAb R3B23 were reconstituted in sodium carbonate buffer (0.05 mol/L, pH 9) and conjugated with *p*-SCN-Bn-CHX-A”-DTPA (Macrocyclics, Plano, TX, USA) for ^111^In-labeling or *p*-SCN-Bn-DOTA (Macrocyclics) for ^225^Ac-labeling. A 15-fold molar excess of bifunctional chelator was incubated with different sdAbs and mAb for 2 h at room temperature. The resulting sdAb conjugates were purified via size-exclusion chromatography (SEC) on a Superdex Peptide 10/300 (GE Healthcare, Eindhoven, The Netherlands) and DTPA-trastuzumab on a Superdex 75 10/30 column (GE Healthcare) and reconstituted in ammonium acetate (0.1 mol/L, pH 7.0).

^111^InCl_3_ was obtained from Mallinckrodt Inc. (Amsterdam, The Netherlands). The desired activity of ^111^In (350–370 MBq) was added to the DTPA conjugates (50–100 µg) in ammonium acetate (0.2 mol/L, pH 5.0) and incubated for 30 min while shaking at 55 °C or room temperature for sdAbs and mAbs respectively while shaking. [^111^In]In-DTPA-2Rs15d and [^111^In]In-DTPA-trastuzumab are referred to as [^111^In]-2Rs15d and [^111^In]-trastuzumab respectively.

^225^Ac was obtained from JRC (Karlsruhe, Germany). The desired activity of ^225^Ac (4.8−6.6 MBq) was added to 100–120 μg of DOTA−2Rs15d conjugate in ammonium acetate buffer (0.5 mol/L, pH 5.2). The mixture was incubated for 90 min at 55 °C while shaking. The reaction mixture was cooled to room temperature. Ten μL of 50 mM DTPA was added to complex unbound ^225^Ac. [^225^Ac]Ac-DOTA-2Rs15d is referred to as [^225^Ac]-2Rs15d.

Purification of all radiolabeled conjugates was performed via NAP-5 SEC (GE Healthcare) with 0.1% Tween −150 mg/mL ascorbic acid in PBS and filtered through a 0.22-mm filter (Millex, Millipore, Darmstadt, Germany). Radiochemical purity was evaluated using instant thin-layer chromatography (iTLC) on silica gel-impregnated glass fiber sheets (Agilent Technologies, Santa Clara, CA, USA) with citrate buffer (0.1 mol/L, pH4.0) used as mobile phase. iTLC read-out of ^225^Ac-labeled compounds was performed 24 h after labeling.

Sodium [^131^I]iodide was obtained from GE Healthcare. Radioiodination of 2Rs15d was performed via the residualizing prosthetic group N-succinimidyl 4- guanodinomethyl-3-[^131^I]benzoate ([^131^I]SGMIB) and purified as reported previously [61]. In short, in a first step BisBoc-SGMIB tin-precursor was radioiodinated, TFA-deprotected and purified via reversed-phase chromatography. In a second step, pure [^131^I]SGMIB was conjugated to 2Rs15d after which the resulting radioconjugate was purified via NAP-5 SEC. iTLC was performed with PBS used as mobile phase. [^131^I]SGMIB-2Rs15d is further referred to as [^131^I]-2Rs15d.

### 4.5. Tumor Inoculation and Follow-Up

Female athymic nude mice (Crl:NU(NCr)-Foxn1^nu^, Charles River, Écully, France; 22 ± 5 g body weight) were inoculated intracranially with 2.5 × 10^5^ SKOV3.IP1 or 231Br cells in 5 µL unsupplemented McCoy’s 5a or DMEM medium, respectively. The cell suspension was injected 2 mm anterior and 2 mm lateral of the bregma at a depth of 1.5 mm using a stereotaxic immobilization frame (World Precision Instruments, Hertfordshire, UK) at a speed of 0.5 µL/min using a UMP3 UltraMicroPump and Micro4 Controller (World Precision Instruments). After tumor inoculation, the burr hole was sealed using bone wax and the skin was sutured. 

Tumor growth was established using in vivo bioluminescence imaging (BLI) (Biospace Lab) after intraperitoneal injection of 150 mg/kg Luciferin [62]. All procedures were performed under 2.5% isoflurane anesthesia (Abbott Laboratories, Lake Bluff, IL, USA).

All animal studies were approved by the Vrije Universiteit Brussel’s Ethical Committee for Animal Testing (16-272-2; 19-272-13). The number of used mice per group for each experiment can be found in their respective Materials and Methods section.

### 4.6. In Vivo Tumor Targeting and Ex Vivo Biodistribution of ^111^In-Labeled Radioconjugates

Mice bearing intracranial HER2^pos^ tumors were intravenously injected with [^111^In]-sdAb or [^111^In]-mAb (28.0–37.0 MBq; *n* = 4/group). One hour and three days post-injection (p.i.), µSPECT/CT imaging was performed with a Vector+/CT MILabs system (MILabs, Utrecht, The Netherlands) under 2.5% isoflurane anesthesia. SPECT-images were obtained using a rat SPECT-collimator (1.5-mm pinholes) in spiral mode, nine positions for whole-body imaging and three positions for brain imaging, with 50 s per position for whole-body imaging and 10 min per position for brain imaging. Images were reconstructed with 0.4 mm^3^ voxels with two subsets and two iterations, without post-reconstruction filter. Image analysis was performed using a Medical Image Data Examiner (AMIDE) software [63].

After imaging, mice were killed, and organs, tissues, and tumors were isolated and weighed. The radioactivity in each sample was measured using a Wizard^2^ γ-counter (PerkinElmer, Waltham, MA, USA). Tracer uptake was expressed as % injected activity per gram organ (%IA/g). Statistical analyses were performed using one-way ANOVA.

### 4.7. Dosimetry Calculations of A Single Dose of [^131^I]- and [^225^Ac]-2Rs15d 

Groups of mice with intracranial 231Br tumors (*n* = 3) were injected i.v. with either 3.67 ± 0.34 MBq [^131^I]-2Rs15d (5.0 μg sdAb) or 65.85 ± 5.25 kBq [^225^Ac]-2Rs15d (5.0 μg sdAb). Mice were killed 1, 4, 12, 24, 48 and 72 h p.i., dissected and organs and tissues were isolated, weighed, counted and expressed as %IA/g. The biodistribution data were time-integrated to calculate the residence time of [^131^I]- and [^225^Ac]-2Rs15d per gram organ. The integration between time 0 and 72 h was made using the trapezoid method. The final 2 points were used to estimate the residence time from 72 h to infinity. For each data set, the absorbed doses were calculated. The S values of ^131^I and ^225^Ac were obtained from RADAR phantoms (www.doseinfo-radar.com/RADARphan.html). The S value for 1 g sphere of 3.04 × 10^−14^ J/Bq.s was used for dose calculations for ^131^I and 4.40 × 10^−12^ J/Bq.s for [^225^Ac] [17].

### 4.8. Theranostic Application of [^131^I]-2Rs15d for Brain Lesions

To assess the therapeutic efficacy of [^131^I]-2Rs15d, intracranial 231Br tumor-bearing mice (*n* = 8/group) received either (i) [^131^I]-2Rs15d (32.37 ± 1.83 MBq; 5 µg sdAb; weekly on D7-14), (ii) trastuzumab (loading dose, 7.5 mg mAb/kg body weight, D7; maintenance dose, biweekly on D10-14-18-21: 3.5 mg mAb/kg body weight), (iii) [^131^I]-2Rs15d + trastuzumab combination treatment (32.37 ± 1.83 MBq; 5 µg sdAb; weekly on D7-14 + trastuzumab regimen [16]), (iv) unlabeled 2Rs15d (5 µg sdAb; weekly on D7-14), or (v) vehicle buffer (0.9% NaCl + 5 mg/mL ascorbic acid). 

Therapy was initiated one week post-tumor inoculation. All treatments were administered intravenously (i.v.) in the tail vein in a total volume of 150 µL. Animals were weighed biweekly and checked daily for general health and wellbeing. Animals were sacrificed when one of the following endpoints was reached: (i) weight loss > 20% of original body weight, (ii) immobility, (iii) unresponsiveness to external stimuli. Survival curves were analyzed using the Log-rank Mantel-Cox test. To evaluate the theranostic potential of [^131^I]-2Rs15d, one additional group of intracranial 231Br tumor-bearing mice (*n* = 3) received [^131^I]-2Rs15d (32.37 ± 1.83 MBq; 5 µg sdAb; weekly on D7-14), identical to the treatment group, after which µSPECT/CT imaging was performed after 2 h using a rat PET-collimator and a spiral scan mode of 94 bed positions (19 s per position). The obtained SPECT-data were reconstructed with a 0.6 mm^3^ voxel size, two subsets and four iterations. Images were analyzed using AMIDE. Uptake of [^131^I]-2Rs15d in organs and tissues was analyzed and expressed as % injected activity per cubic centimeter (%IA/cc). 

### 4.9. Targeted Alpha Therapy of Brain Lesions

In the first therapy experiment, intracranial 231Br tumor-bearing mice (*n* = 8/group) received either (i) [^225^Ac]-2Rs15d (81.67 ± 28.87 kBq; 5 µg sdAb; weekly on D7-14-21), (ii) trastuzumab (loading dose, 7.5 mg mAb/kg body weight, D7; maintenance dose, biweekly on D10-14-18-21: 3.5 mg mAb/kg body weight) (iii) [^225^Ac]-2Rs15d + trastuzumab combination treatment (81.67 ± 28.87 kBq; 5 µg sdAb; weekly on D7-14-21 + trastuzumab regimen), or (iv) vehicle buffer (0.9% NaCl + 5 mg/mL ascorbic acid). [^225^Ac]-2Rs15d was co-administered with 150 mg/kg gelofusin as previously optimized in order to reduce kidney retention [20].

In the second therapy experiment, intracranial SKOV3.IP1 tumor-bearing mice (*n* = 8/group) received either (i) [^225^Ac]-2Rs15d (85.33 ± 25.25 kBq; 5 µg sdAb; weekly on D7-14-21), (ii) trastuzumab regimen (iii) [^225^Ac]-2Rs15d + trastuzumab combination treatment (85.33 ± 25.25 kBq; 5 µg sdAb; weekly on D7-14-21 + trastuzumab regimen), or (iv) vehicle buffer (0.9% NaCl + 5 mg/mL ascorbic acid). [^225^Ac]-2Rs15d was co-administered with 150 mg/kg gelofusin as previously optimized in order to reduce kidney retention.

Therapy started one week post-tumor inoculation. All treatments were administered i.v. in the tail vein in a total volume of 150 µL. Animals were weighed biweekly and checked daily for general health and wellbeing. Animals were sacrificed when one of the following endpoints was reached: (i) weight loss > 20% of original body weight, (ii) immobility, (iii) unresponsiveness to external stimuli. Survival curves were analyzed using the Log-rank Mantel-Cox test.

### 4.10. Toxicity of [^225^Ac]-2Rs15d and [^131^I]-2Rs15d

During targeted α- and β^−^-radionuclide therapy experiments, mice that reached one of the experimental endpoints were sacrificed and liver, spleen, kidneys, heart, brain and lung were isolated, fixed in formalin, and embedded in paraffin wax. The samples were trimmed and processed by AnaPath Services GmbH (Liestal, Switzerland). Sections of 4 µm thickness were taken and stained with haematoxylin and eosin. The sections were quality-assessed and examined by light microscopy by qualified pathologists. Toxicity to tissues was compared between vehicle buffer treatment and ^131^I- or ^225^Ac-labeled sdAbs. 

## 5. Conclusions

As systemic treatments have improved over the years, it has become clear that the incidence of brain metastases steadily increases, and that control of CNS disease is becoming more important. We have demonstrated that radiolabeled sdAbs are ideal vehicles for targeted radionuclide therapy and molecular imaging, not only for systemic disease, but also for metastatic lesions in the brain. Moreover, histopathological analysis after therapy revealed no significant early toxicity.

## Figures and Tables

**Table 1:** Ex Vivo biodistribution of [^131^I]-2Rs15d and corresponding absorbed dose after i.v. injection of 37MBq [^131^I]-2Rs15d in HER2^pos^ 231Br tumor-bearing mice (*n* = 3 per time point).

Ex Vivo Biodistribution																			Dosimetry
Organ	1 h p.i.			4 h p.i.			12 h p.i.			24 h p.i.			48 h p.i.			72 h p.i.			Absorbed
	Mean		SD	Mean		SD	Mean		SD	Mean		SD	Mean		SD	Mean		SD	Dose
Brain	0.09	±	0.03	0.05	±	0.03	0.03	±	0.01	0.01	±	0.00	0.00	±	0.00	0.00	±	0.00	**0.04**
Lungs	0.73	±	0.25	0.36	±	0.12	0.13	±	0.02	0.08	±	0.02	0.03	±	0.01	0.02	±	0.00	**0.27**
Heart	0.53	±	0.11	0.29	±	0.12	0.16	±	0.02	0.01	±	0.00	0.01	±	0.00	0.00	±	0.00	**0.18**
Liver	1.55	±	0.23	0.64	±	0.16	0.19	±	0.07	0.08	±	0.01	0.06	±	0.01	0.01	±	0.00	**0.43**
Kidneys	62.63	±	9.54	19.20	±	3.57	6.59	±	2.46	1.88	±	0.52	0.84	±	0.23	0.44	±	0.02	**12.50**
Spleen	0.33	±	0.10	0.19	±	0.10	0.05	±	0.02	0.01	±	0.00	0.01	±	0.00	0.00	±	0.00	**0.10**
Muscle	0.54	±	0.09	0.29	±	0.05	0.10	±	0.03	0.05	±	0.01	0.03	±	0.01	0.01	±	0.00	**0.21**
Bone	0.46	±	0.08	0.20	±	0.09	0.11	±	0.04	0.05	±	0.01	0.02	±	0.01	0.01	±	0.00	**0.18**
Small intestines	0.50	±	0.14	0.42	±	0.04	0.09	±	0.02	0.01	±	0.00	0.01	±	0.00	0.00	±	0.00	**0.17**
Large intestines	0.44	±	0.19	0.23	±	0.10	0.18	±	0.06	0.02	±	0.01	0.01	±	0.00	0.01	±	0.00	**0.18**
Blood	0.86	±	0.14	0.31	±	0.08	0.05	±	0.01	0.01	±	0.00	0.01	±	0.00	0.00	±	0.00	**0.16**
Thyroid	2.01	±	0.93	1.97	±	0.27	1.56	±	0.77	0.91	±	0.00	0.76	±	0.25	0.52	±	0.02	**3.99**
Tumor	4.51	±	2.55	4.81	±	2.49	3.54	±	2.01	1.89	±	1.90	0.62	±	0.32	0.29	±	0.01	**4.90**

Biodistribution data points represent an average ± SD and are expressed as %IA/g. Dosimetry data are expressed as Gy per 37MBq.

**Table 2:** Ex Vivo biodistribution of [^225^Ac]-2Rs15d and corresponding absorbed dose after i.v. injection of 85 kBq [^225^Ac]-2Rs15d in HER2^pos^ 231Br tumor-bearing mice (*n* = 3 per time point).

Ex Vivo Biodistribution																			Dosimetry
Organ	1 h p.i.			4 h p.i.			12 h p.i.			24 h p.i.			48 h p.i.			72 h p.i.			Absorbed
	Mean		SD	Mean		SD	Mean		SD	Mean		SD	Mean		SD	Mean		SD	Dose
Brain	0.07	±	0.03	0.06	±	0.02	0.03	±	0.01	0.01	±	0.00	0.00	±	0.00	0.00	±	0.00	**0.02**
Lungs	0.69	±	0.22	0.34	±	0.09	0.11	±	0.02	0.04	±	0.02	0.01	±	0.01	0.01	±	0.00	**0.08**
Heart	0.49	±	0.10	0.36	±	0.09	0.16	±	0.02	0.01	±	0.00	0.01	±	0.00	0.00	±	0.00	**0.07**
Liver	1.15	±	0.20	0.71	±	0.12	0.13	±	0.06	0.02	±	0.01	0.04	±	0.01	0.01	±	0.00	**0.12**
Kidneys	32.03	±	8.49	16.30	±	2.78	5.79	±	2.09	2.65	±	0.44	0.53	±	0.25	0.23	±	0.02	**3.49**
Spleen	0.36	±	0.09	0.13	±	0.08	0.05	±	0.02	0.01	±	0.00	0.01	±	0.00	0.00	±	0.00	**0.03**
Muscle	0.24	±	0.08	0.21	±	0.04	0.12	±	0.03	0.04	±	0.01	0.01	±	0.01	0.01	±	0.00	**0.06**
Bone	0.38	±	0.07	0.22	±	0.07	0.17	±	0.03	0.03	±	0.01	0.02	±	0.01	0.01	±	0.00	**0.07**
Small intestines	0.57	±	0.12	0.34	±	0.03	0.04	±	0.02	0.02	±	0.00	0.01	±	0.00	0.00	±	0.00	**0.05**
Large intestines	0.49	±	0.17	0.23	±	0.08	0.15	±	0.05	0.04	±	0.01	0.01	±	0.00	0.01	±	0.00	**0.07**
Blood	0.73	±	0.12	0.25	±	0.06	0.10	±	0.01	0.02	±	0.00	0.01	±	0.00	0.00	±	0.00	**0.06**
Thyroid	0.00	±	0.83	0.00	±	0.21	0.00	±	0.65	0.00	±	0.00	0.00	±	0.28	0.00	±	0.03	**0.00**
Tumor	3.81	±	1.13	4.01	±	1.08	3.54	±	1.08	1.39	±	0.89	0.62	±	0.15	0.44	±	0.01	**1.47**

Biodistribution data points represent an average ± SD and are expressed as %IA/g. Dosimetry data are expressed as Gy per 85 kBq.

## Supplementary Materials

The following are available online at https://www.mdpi.com/2072-6694/12/4/1017/s1, Figure S1: Treatment regime for the theranostic application of [^131^I]-2Rs15d for brain lesions, Figure S2: Treatment regime for targeted alpha therapy of brain lesions, Table S1: Post-treatment incidence and mean severity of toxicity.
