# Supplementary material for: Preclinical Targeted α- and β−-Radionuclide Therapy in HER2-Positive Brain Metastasis Using Camelid Single-Domain Antibodies
Source: Cancers (Basel). 2020 Apr 21;12(4):1017. doi: 10.3390/cancers12041017 (PMC7226418; doi:10.3390/cancers12041017)
Supplement: Supplementary file 1 [file cancers-12-01017-s001.pdf]

Supplementary Materials:

Preclinical Targeted α- and β−-Radionuclide Therapy in HER2-Positive Brain Metastasis Using Camelid Single-Domain Antibodies

Janik Puttemans, Yana Dekempeneer, Jos L. Eersels, Heleen Hanssens, Pieterjan Debie, Marleen Keyaerts, Albert D. Windhorst, Frank van der Aa, Quentin Lecocq, Karine Breckpot, Alfred Morgenstern, Frank Bruchertseifer, Tony Lahoutte, Nick Devoogdt and Matthias D'Huyvetter

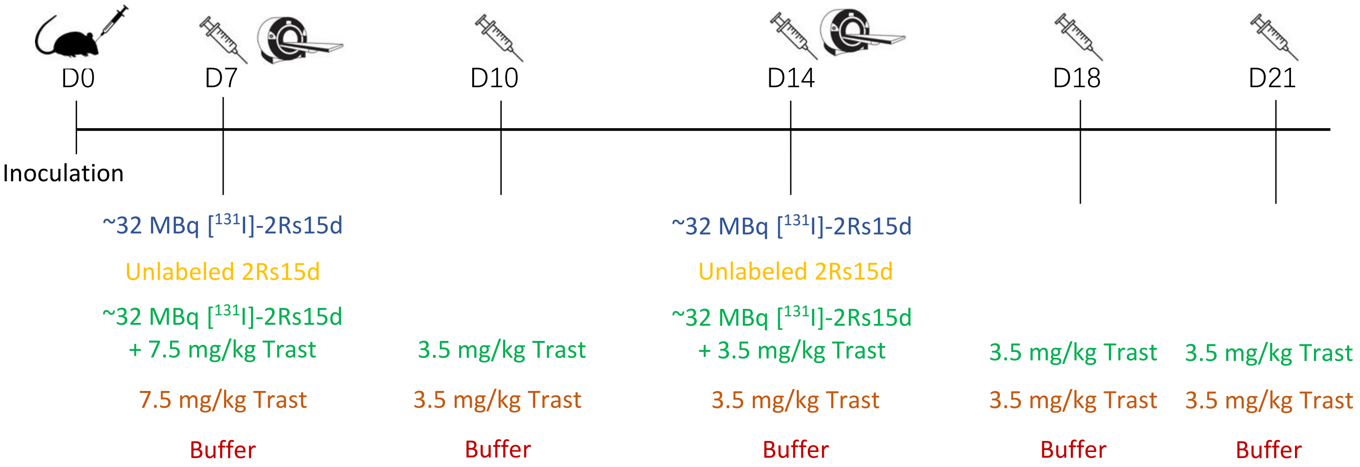

Figure S1. Treatment regime for the theranostic application of [^{131}I]-2Rs15d for brain lesions
Intracranial 231Br tumor-bearing mice (n=8/group) received either (i) [^{131}I]-2Rs15d (32.37 ± 1.83 MBq; 5 µg sdAb; weekly on D7-14), (ii) Trastuzumab (loading dose, 7.5 mg mAb/kg body weight, D7; maintenance dose, biweekly on D10-14-18: 3.5 mg mAb/kg body weight), (iii) [^{131}I]-2Rs15d + Trastuzumab combination treatment (32.37 ± 1.83 MBq; 5 µg sdAb; weekly on D7-14 + Trastuzumab regimen [16]), (iv) unlabeled 2Rs15d (5 µg sdAb; weekly on D7-14), or (v) vehicle buffer (0.9% NaCl + 5 mg/mL ascorbic acid). Therapy was initiated one week post-tumor inoculation. All treatments were administered intravenously (i.v.,) in the tail vein in a total volume of 150 µL.
To evaluate the theranostic potential of [^{131}I]-2Rs15d, one additional group of intracranial 231Br tumor-bearing mice (n=3) received [^{131}I]-2Rs15d (32.37 ± 1.83 MBq; 5 µg sdAb; weekly on D7-14), after which µSPECT/CT imaging ()was performed 2 h post-injection.

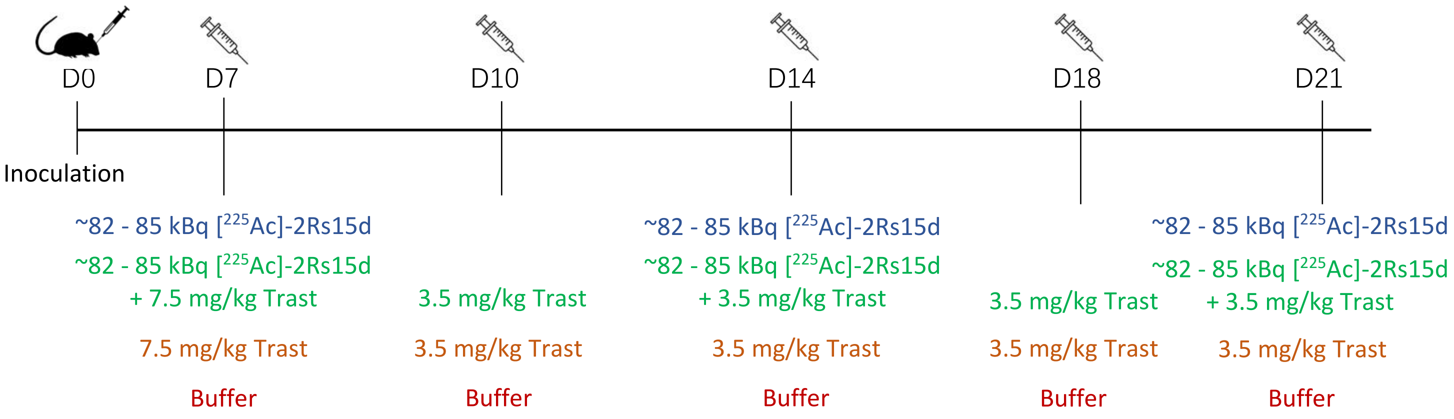

Figure S2. Treatment regime for targeted alpha therapy of brain lesions
Intracranial 231Br or SKOV3.IP1 tumor-bearing mice (n=8/group) received either (i) [^{225}Ac]-2Rs15d (81.67 ± 28.87 kBq for 231Br tumors, 85.33 ± 25.25 kBq for SKOV3.IP1; 5 µg sdAb; weekly on D7-14-21), (ii) Trastuzumab (loading dose, 7.5 mg mAb/kg body weight, D7; maintenance dose, biweekly on D10-14-18-21: 3.5 mg mAb/kg body weight) (iii) [^{225}Ac]-2Rs15d + Trastuzumab combination treatment (81.67 ± 28.87 kBq; 5 µg sdAb; weekly on D7-14-21 + Trastuzumab regimen), (iv) unlabeled 2Rs15d (5 µg sdAb; weekly on D7-14-21), or (v) vehicle buffer (0.9% NaCl + 5 mg/mL ascorbic acid). [^{225}Ac]-2Rs15d was co-administered with 150 mg/kg Gelofusin as previously optimized in order to reduce kidney retention. Therapy started one week post-tumor inoculation. All treatments were administered i.v. ( ) in the tail vein in a total volume of 150 µL.

Table S1: Post-treatment incidence and mean severity of toxicity

	Non-treated	[^{131}I]-2Rs15d	[^{131}I]-2Rs15d + Trast	[^{225}Ac]-2Rs15d	[^{225}Ac]-2Rs15d + Trast
**Kidneys**					
Tubular basophilia	1/1.0	1/1.0	1/1.0	1/2.0	0
Tubular dilation	0	1/3.0	0	2/2.5	2/3.0
Cytoplasmic vacuoles	3/1.0	3/1.5	2/1.5	3/1.5	2/1.0
**Heart**					
Inflammation	0	2/1.0	1/1.0	0	0
Myocardial necrosis	0	2/1.5	0	0	0
Intracytoplasmic vacuolation	0	2/1.5	1/1.0	0	0
**Lung**					
Epithelium necrosis	2/1.0	1/1.0	0	0	2/1.0
Haemorrhage	1/1.0	0	1/1.0	0	0
Atelectasis	2/1.5	1/1.0	0	2/1.0	0

Values are represented as number of mice presented with symptoms/degree of symptoms.
Histological changes were described according to distribution, severity and morphologic character. Severity scores were assigned from a scale of 1-5.

Grade 1, Minimal	This corresponds to a histopathologic change ranging from inconspicuous to barely noticeable but so minor, small, or infrequent as to warrant no more than the least assignable grade. For multifocal or diffusely distributed lesions, this grade was used for processes where less than approximately 10 % of the tissue in an average high-power field was involved.
Grade 2, Slight	This corresponds to a histopathologic change that is a noticeable but not a prominent feature of the tissue. For multifocal or diffusely distributed lesions, this grade was used for processes where between approximately 10% and 25% of the tissue in an average high-power field was involved.
Grade 3, Mild	This corresponds to a histopathologic change that is a prominent but not a dominant feature of the tissue. For multifocal or diffusely distributed lesions, this grade was used for processes where between approximately 25% and 50% of the tissue in an average high-power field was involved.
Grade 4, Moderate	This corresponds to a histopathologic change that is a dominant but not an overwhelming feature of the tissue. For multifocal or diffusely distributed lesions, this grade was used for processes where between approximately 50% and 95% of the tissue in an average high-power field was involved.
Grade 5, Severe	This corresponds to a histopathologic change that is an overwhelming feature of the tissue. For multifocal or diffusely distributed lesions, this grade was used for processes where greater than approximately 95% of the tissue in an average high-power field was involved.

© 2020 by the authors. Submitted for possible open access publication under the terms and conditions of the Creative Commons Attribution (CC BY) license (http://creativecommons.org/licenses/by/4.0/).
